# 2703. Descriptive Epidemiology of Respiratory Syncytial Virus (RSV) Infection – a 10-year Review at the National Institutes of Health (NIH) Clinical Center

**DOI:** 10.1093/ofid/ofad500.2314

**Published:** 2023-11-27

**Authors:** LaToya A Forrester, Robin T Odom, David K Henderson, Ninet Sinaii

**Affiliations:** National Institutes of Health Clinical Center, Bethesda, Maryland; NIH Clinical Center, Jefferson, Maryland; NIH, Bethesda, MD; National Institutes of Health, Bethesda, Maryland

## Abstract

**Background:**

RSV can lead to severe infection in immunocompromised individuals. We performed a 10-year retrospective review of RSV infections to assess the pre-COVID-19 pandemic versus current RSV epidemiology. Asymptomatic patients were tested for RSV to clear isolation precautions and inadvertently, when testing for SARS-CoV-2 prior to aerosol–generating procedures or hospital admission.

**Methods:**

Nasopharyngeal swabs and washes, bronchoalveolar lavages, and bronchial washes collected 1/2012-12/2022 were tested by multiplex PCR. Chart reviews were completed for RSV-positive patients to detect respiratory symptoms on admission through day 8 to delineate community- vs. healthcare-acquired cases, determine comorbidities, identify co-infection with other respiratory viruses, and stem-cell transplant recipients within the prior year. Data were analyzed using longitudinal models.

**Results:**

Overall 17,023 respiratory tests were sent during the 10-year period, with 537 RSV-positives from 286 patients, a 3.15% positivity. A decrease in RSV detection was observed between the pre-pandemic and pandemic time periods for all patients (p=0.007) and for adults (p=0.011). Cases were community-acquired (85% in pediatrics and 95% in adults). The rationale for testing was symptom-based for 82% of pediatrics and 91% of adults. Positive tests were observed in pediatrics with congenital immune deficiency diseases (53%) and in adults with hematologic malignancy (44%). 35% of pediatrics and 42% of adults had a stem-cell transplant within the prior year. 21% of pediatrics and 20% of adults were co-infected with atleast one other respiratory virus. Incidental screening of asymptomatic patients identified RSV in 24 patients. Cyclical changes in RSV occurred during the pre-pandemic years, and a substantial decrease and irregularity (p=0.007) were noted in the monthly frequency of RSV beginning in 2020.

**Conclusion:**

RSV remains a common pathogen in immunocompromised patients. Persistent RSV positivity was identified in the absence of symptoms and after treatment for RSV infection. We observed a sharp decrease in RSV activity in 2020; the subsequent gradual increase has not reached pre-pandemic levels.

**Disclosures:**

**All Authors**: No reported disclosures

